# Acquired Sleep-Related Hypermotor Epilepsy with Disrupted White Matter Tracts Assessed by Multishell Diffusion Magnetic Resonance Imaging

**DOI:** 10.3389/fneur.2018.00006

**Published:** 2018-01-22

**Authors:** Zahari N. Tchopev, Ping-Hong Yeh, Greg W. Morgan, Eric Meyer, Johanna M. Wolf, John M. Ollinger, Gerard P. Riedy, Lisa C. Young

**Affiliations:** ^1^School of Medicine, Uniformed Services University of the Health Science, Bethesda, MD, United States; ^2^National Intrepid Center of Excellence, Walter Reed National Military Medical Center, Bethesda, MD, United States; ^3^Department of Psychiatry, Walter Reed National Military Medical Center, Bethesda, MD, United States; ^4^Inpatient Neurobehavioral Program, Walter Reed National Military Medical Center, Bethesda, MD, United States

**Keywords:** sleep-related hypermotor epilepsy, traumatic brain injury, magnetic resonance imaging, tractography, EEG, posttraumatic epilepsy

## Abstract

Sleep-related hypermotor epilepsy (SHE) (previously frontal lobe epilepsy) is a rare seizure disorder commonly misdiagnosed or unrecognized, causing negative patient sequelae. While usually reported in familial studies, it is more commonly acquired. Diagnosis is a challenge due to its low incidence in comparison with the more common sleep disorders or psychogenic etiologies in the differential diagnosis. Diagnosis is scaled on degree of certainty based on described or clinically documented semiology, with video EEG as a helpful, but not necessary, adjunct. Current treatment is similar to other focal epilepsies. We studied a 36-year-old active duty male soldier who presented with 2 years of predominantly sleep related, abrupt, short, and anamnestic hyperkinetic movements with unstructured vocalizations. Prior workup included non-contributory video electroencephalograph (EEG) and polysomnography as well as normal brain magnetic resonance imaging (MRI). Treatments for presumed psychiatric and parasomnia disturbances were not effective in establishing diagnosis or relief. Evaluation at our tertiary, multidisciplinary care institution recorded events consistent with the diagnosis of clinical SHE. He was enrolled in an advanced multishell diffusion-weighted imaging MRI research study to evaluate white matter tracts, given his history of mild, repetitive, non-penetrating traumatic brain injury, not otherwise requiring hospitalization. Multishell diffusion MRI tractography found changes not previously described in the right frontal lobe white matter tracts. These changes were consistent with neurological localization and serve as a potential nidus for this patient’s seizure disorder. Misdiagnosis of SHE can result in detrimental biopsychosocial sequelae of untreated epilepsy, unnecessary or harmful intervention, or the stigmata of a behavioral disorder. Further investigation into diagnosis and etiology of acquired SHE is needed. Assessment for white matter abnormalities can potentially provide information into pathogenesis of epilepsy disorders.

## Introduction

The following case study illustrates the difficulties in diagnosis of a rare disease in the setting of atypical report of worsening signs and symptoms, the benefits of multidisciplinary diagnostic approach, and limitations individual providers may have in diagnostic confidence. We review the most up-to-date diagnostic criteria, proposed etiologies and treatments for sleep-related hypermotor epilepsy (SHE). To the best of our knowledge, this is first case report which has localized this rare epileptic disorder with both video encephalographic and structural imaging changes by diffusion magnetic resonance imaging (MRI).

Sleep-related hypermotor epilepsy was first described in a handful of patients from Bologna, Italy in 1981 (1). Characterized by bizarre movements and posturing occurring from sleep in the absence of electroencephalograph (EEG) activity, it was labeled “hypnogenic paroxysmal dystonia,” and modified some time later to “nocturnal paroxysmal dystonia” (1, 2). When the semiologic similarity was noted in patients with EEG recorded frontal seizures undergoing neurosurgical intervention for drug-resistant epilepsy, the name was changed to nocturnal frontal lobe epilepsy (3). Finally, in 2014, FNLE was renamed to the present SHE by a group of international experts in epilepsy and sleep with the intent of facilitating more accurate diagnosis and recognition (4).

Sleep-related hypermotor epilepsy has an estimated prevalence of 1.8/100,000, classifying it as a rare disease (5). However, this incidence rate is as reported in Italy, where there is a higher concentration of SHE due to autosomal dominant inheritance. The true worldwide acquired incidence is likely lower, although not reported in the literature, as further population level studies are necessary. Low or unknown prevalence in a relatively newly described disease likely contributes to lack of international diagnostic criteria and thus, delayed recognition and misdiagnosis.

Diagnosis is based primarily on clinical history. The seizures are typically stereotyped motor patterns with abrupt onset, last briefly (<2 min), and abruptly stop. Clustering is common, but not required. The most common motor phenotype is one with vigorous or bizarre hyperkinetic features while seizures with asymmetric tonic or dystonic posturing is the second most common presentation. The former is often described or recorded as kicking, pedaling, grasping attempts or rolling, and rocking movements. A minor characteristic may include unstructured vocalization (screaming) (6). Patients may report being aware of the episode but not being able to control the behavior, as well as abrupt arousal or distinct aura. Occasionally, a patient may describe jumping up and running, a phenomenon named Epileptic Nocturnal Wandering (ENW) (7). While occurring predominantly during sleep, they may also occur in wakefulness. Notably, diagnosis is not excluded by absence of ictal EEG correlates or even extrafrontal origin (extrafrontal in up to 30%) (8, 9). This is often because ictal EEG is obscured by artifacts of the patient’s violent movements and thus renders the data uninformative. In cases where EEG is collected, seizures generally occur during the slow wave phase of non-rapid eye movement sleep (NREM) (10, 11). Provider certainty of SHE diagnosis is categorized as witnessed (possible), video documented (clinical) and video-EEG documented (confirmed), whereby the semiology is either only reported, video recorded, or correlated to EEG, respectively.

The etiology and anatomo-electroclinical correlations are under further elucidation. In the majority of cases, etiology is unknown. Identified etiologies may include focal cortical dysplasia, acquired injuries or genetic (10, 12–14). Genetic studies have elucidated heterogeneous mutations to include the nicotinic acetylcholine receptor and sodium-activated potassium channels (15, 16). Furthermore, functional studies suggest hyperactivation of the cholinergic pathway, perhaps elucidating a pathophysiology, as this pathway modulates both sleep and arousal (17). Providers evaluating vEEG can consider involvement of the mesialdorsolateral, orbitopolar, opercular, or larger lobar cortical regions as sources of the hyperkinetic behavior. The fear-associated behaviors of ENW, or screaming, implicate structures of the limbic system, temporal region, and anterior cingulate (18–20).

When identified, SHE requires prompt treatment and education on seizure precautions. Treatment options are similar to other focal epilepsies as pathophysiology is better elucidated over time. Carbamazepine has been reported as the drug of choice, although others have been shown to be effective (4). All patients should be advised on appropriate restrictions and precautions, often based on state of residence, to prevent injury. As sleep alteration can be a comorbid disorder and contribute to seizure precipitation, patients should be instructed to adhere to good sleep hygiene and evaluated for obstructive sleep apnea (21, 22).

## Case Summary

The patient is a 36-year-old man with a 2-year history of bizarre, hypermotor spells predominantly at nighttime admitted to the Neurobehavioral and traumatic brain injury (TBI) Unit of Walter Reed National Military Medical Center (WRNMMC). History of his illness was corroborated by his wife *via* telephone. The reported spells occurred at a rate range of 3–5 times while awake and up to 7–9 times from sleep. Sleep deprivation was perceived to increase rate of nighttime episodes. The subject described occasional “warming” aura before episodes and intense fatigue after episodes, which last 10–15 s, stopping and ending abruptly. When awake, episodes are described as a yell or grunting and jumping up or running. The subject used phrases such as “fight or flight sensation” and “panic.” At night, episodes were described as a sudden sitting-up, yelling, and jumping out of bed in dramatic fashion, with uncomplicated return to sleep afterward. Episodes were anamnestic to the patient. He endorsed exceptionally rare associated loss of bladder control. He denied self-awareness and control at initiation of an episode but described reorientation and redirection of his actions toward the end, such as patting someone on the back when running past them or, offering a “high-five.” He further denied temporally related oral injuries, changes in cognition, strength, sensation, and preceding olfactory hallucination. He had never injured others during episodes although had injured himself when leaping out of bed. He denied associated nightmares but does still dream, although more rarely due to sleep interruption.

He has no pertinent past medical or surgical history. Admission medications included venlafaxine 225 mg and propranolol 40 mg, prescribed by primary care and behavioral health providers to treat presumed behavioral disorder. Previous related medication trials included paroxetine, bupropion, sertraline, hydroxyzine, trazodone, and clonazepam. He completed behavioral health therapy for psychiatric and sleep-related diagnoses. No hypermotor episode frequency change was appreciated by the patient or spouse in that time. His family history is negative for movement disorders and parasomnias in his parents, but he has a sister with resolved juvenile myoclonic epilepsy. On review of systems, he endorsed changes in mood and energy, which he attributed to the impact his spells were having on his quality of life. He reported mild, recurrent traumatic brain injury history. He estimated one to two sports related concussions, several head impacts and a single temporary loss of consciousness. Later, during military training exercises and three deployments as an active duty military officer, he was exposed in close proximity to the regular discharge of weapons and ordnance, to include at least five high impact aerial landings, causing some degree of disorientation. He denied the persistence of somatic symptoms after these events. He suffered an indirect blast wave in 2007, causing disorientation, confusion, and epistaxis. He did not seek medical workup or require hospitalization after any of these events. He denied similar head injury for at least 2 years before onset of these symptoms. Lack of *a posteriori* knowledge of TBI significance likely contributed to difficulties in more precise description.

Workup before our evaluation was appropriate. Outside brain MRI was read as normal and revealed no intracranial findings. A sleep study with vEEG and polysomnography (PSG) was interpreted as periodic limb movements/REM behavior disorder and sleep apnea with insomnia. A 24-h ambulatory EEG with event recorder reported normal background during all episodes. As a result, overall, previous pharmacologic and non-pharmacologic treatment was directed toward behavioral health and sleep-related disorders.

**Figure 1:**
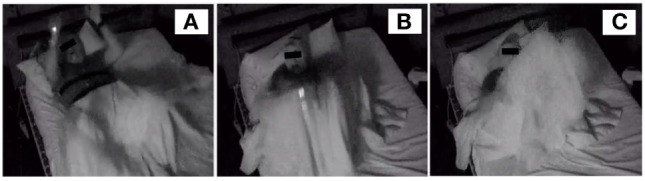
Video EEG screenshots of sleep-related hypermotor epilepsy patient. **(A)** Bilateral hand raising, leg flexion, and moaning. **(B)** Yelling and abrupt sit-up. **(C)** Forceful lay back and bilateral pedal kicking.

Our admission physical exam and screening labs were within normal limits. Post-spell lactate was normal. He was tapered off of venlafaxine and propranolol without changes in episode rate or mood. He scored unremarkably on psychometric testing. Further review of parasomnia and psychiatric questioning was without significant positive findings. Sleep vEEG and PSG was repeated. The patient was observed to have five episodes with stereotyped behaviors arousing out of drowsiness, stage 2 and stage 3 of non-rapid eye movement (REM) sleep. Behaviors included bilateral flexing of his legs and kicking feet, flexing arms, moaning loudly, sitting up in bed, forcefully flinging back down and suddenly standing up out of bed (Figure 1; Video S1 in Supplementary Material). He was able to immediately answer the sleep technologist over the intercom. Duration of behaviors was about 15 s long without interpretable ictal EEG correlates, secondary to movement noise. There were a few right frontal epileptiform sharp transients that phase reversed on interictal EEG at probe F8 (right frontal). Ictal EEG waveforms were non-diagnostic due to severe movement artifact (Figure S1 in Supplementary Material). Moderate obstructive sleep apnea was also described.

Video EEG allowed for the semiologic diagnosis of SHE. He was initiated on and titrated to a therapeutic dose of oxcarbazepine, a functional relative of carbamazepine that lacks the complications of autoinduction (23). Seizures and related activity were completely resolved after 24 h at a dose of 1,800 mg/day. The patient was discharged and self-reports continued control of seizures 3 months later.

**Figure 2:**
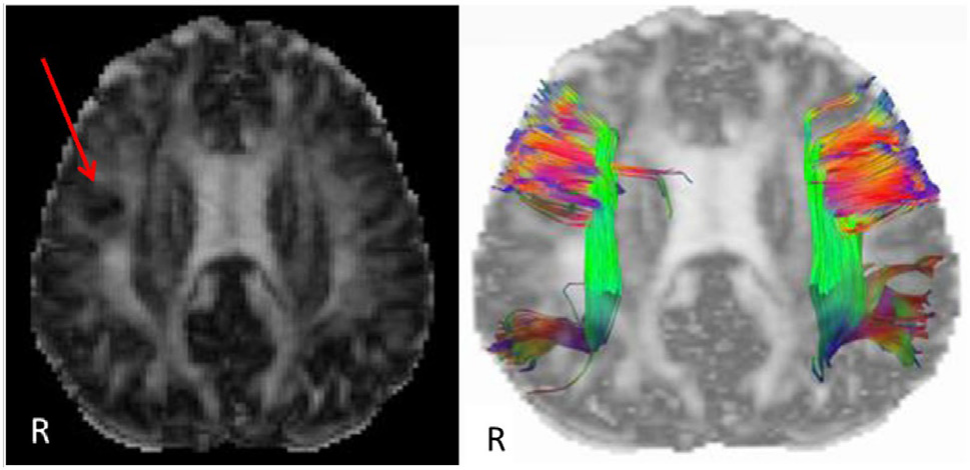
Reconstructed generalized fractional anisotropy (gFA) (left) of subject with a red arrow pointing to white matter disruption with relatively lower gFA of the right frontal lobe. A tractogram overlaid on gFA (right) of the superior longitudinal fasciculus shows sparser tract density on the right compared with left side. Colors indicate fiber tract orientation, or directionality, such that green is anterior–posterior, red is left–right, and blue is superior–inferior.

**Figure 3:**
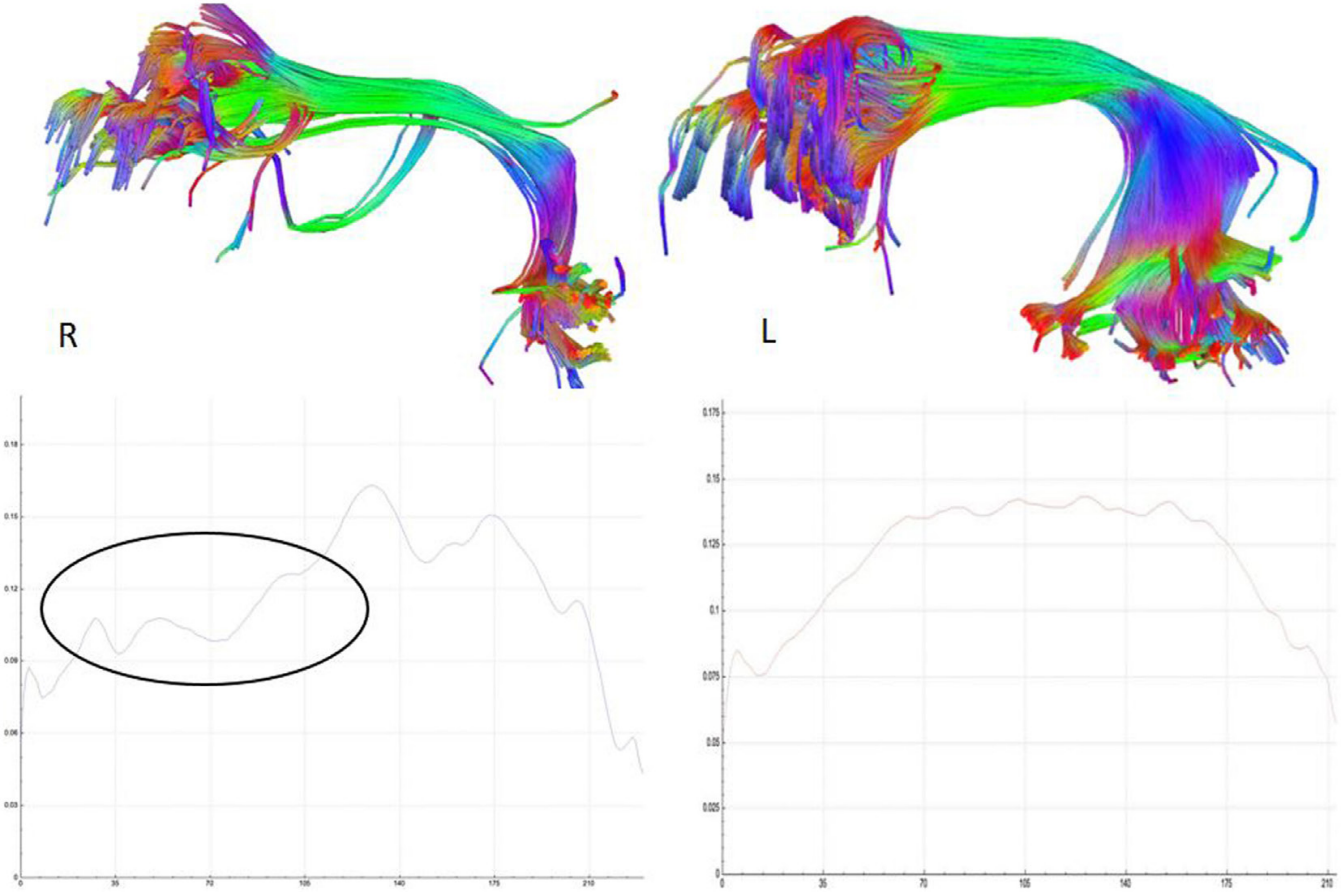
Reconstructed right and left, respectively, superior longitudinal fasciculus (SLF) tracts (above) and their corresponding average generalized fractional anisotropy (gFA) values along the tract plotted below. The gFA profile plots show lower values (circled) over the anterior segment of SLF on the right compared with left, indicating white matter tract damage.

Etiology in this subject was unlikely to be genetic given the autosomal dominant pattern of SHE presenting earlier in life. He had a relatively benign developmental and family history, leaving an acquired etiology as a possible explanation. The patient was voluntarily enrolled in an existing approved study designed to use multishell diffusion-weighted imaging (DWI) MRI and tractography in brain injured patients to evaluate for diffuse axonal injury (DAI), not otherwise appreciated on traditional imaging techniques, such as computed tomography or outpatient MRI. Furthermore, tractography has been shown to detect significant white matter abnormalities in various non-lesional epilepsy syndromes, despite epilepsy being classically considered a disease of gray matter (24–26). This study was carried out in accordance with the recommendations of WRNMMC Institutional Review Board (20337) and Department of Defense, with written informed consent from the subject to participate and for his case and results, including supplementary videos and images, to be published in a scientific journal. The subject gave written informed consent in accordance with the Declaration of Helsinki. A multishell diffusion scheme was used, with *b*-values of 1,000, 2,000, and 3,000 s/mm^2^ and additional 19 volumes of non-DWI, and resolution of 1.7 mm × 1.7 mm × 1.7 mm. Each *b*-value was sampled in 90° directions. Two sets of DWI with opposite phase-encoding directions were acquired using simultaneous multislice sequence with a 3.0 T GE scanner accompanied by 32-channel head coil. The diffusion data were reconstructed using generalized q-sampling imaging (GQI) after preprocessing steps including denoising and correction of geometric distortion (27–29). As had previously been reported in this patient, no findings were appreciated on traditional structural MRI. However, using multishell GQI tractography, the right superior longitudinal fasciculus (SLF) was noted to be damaged, as evidence by a lesion in the image itself, as well as decreased generalized fractional anisotropy (gFA) compared with the contralateral SLF (Figures 2 and 3).

## Discussion

Sleep-related hypermotor epilepsy can be frequently misdiagnosed. It is commonly mistaken for NREM arousal disorders (i.e., sleepwalking and sleep terrors), REM sleep disorders, sleep-related movement disorders, or other nocturnal events. Common psychiatric misdiagnoses include psychogenic non-epileptic seizures (pseudoseizures) and panic attacks, or inappropriately associated with other psychiatric disorder. Diagnostic scales and interviews have been designed to aid in diagnoses and differentiation from parasomnia, however, not from psychiatric disorder (6, 30). Use of these scales presumes a wide enough differential diagnosis and clinical experience of the provider to initiate appropriate workup.

Negative consequences for patients include living with untreated seizures and all their associated sequelae, while others undergo expensive, unnecessary or harmful treatment, while living with the stigma of a behavioral illness. Providers at all levels can help these patients and refer for epilepsy evaluation and treatment. The primary care provider can recognize the abnormal movements and seizure spell characteristics as unlike any traditional disease script. Sleep medicine physicians can appreciate stereotyped, bizarre bilateral movements on vEEG. Psychiatrists can rule out or treat concomitant disorders.

This patient was diagnosed using the semiology from clinically documented video EEG. This is the second highest level of certainty, as per the most up-to-date consensus criteria (4). He also responded to the appropriate pharmacologic treatment for this disorder. The findings of right frontal epileptiform sharp transients that phase reverse are a signature of seizure focus (31). Probe F8 associates with Brodmann area 45R, part of the pars triangularis of the right inferior frontal gyrus (32). This area’s function has been shown to localize to inhibition and impulse control (33). TBI may cause DAI not observable by traditional imaging. Sequelae of the toxic metabolic cascade result in degeneration of neuronal pathways, measurable years beyond initial injury (34–37). A decreased axonal density results in measurable changes in water molecule diffusion properties along the direction of white matter tracts. In this patient, the SLF was damaged. It localizes to regulation of motor behavior, spatial attention, and language. There are four components of SLF, namely, SLF I, II, III, and the arcuate fasciculus (38). The ventral component, interconnecting the ventral prefrontal cortex and the inferior parietal lobule, and possible connection between the pars opercularis, triangularis, and supramarginal gyrus, may function to transfer somatosensory information such as language articulation, and to monitor orofacial and hand motions (39). In humans, right hemispheric dominance, particularly the SLF II and the SLF III, has been suggested to be associated with an unbalanced speed of visuospatial processing (40). A much sparser fiber density and lower values of gFA over the right SLF in this patient suggests non-lesional white matter disruption, which may be related to hyperkinetic behavior, as well as possible impaired visuospatial function. Overall, EEG and imaging findings found pathological changes to the right frontal lobe. The postulated mechanism of this acquired case of SHE may be similar to that of posttraumatic epilepsy (PTE), with the aforementioned lesion serving as a seizure nidus. PTE is a described phenomenon in which TBI patients develop epilepsy years after insult, similar to this patient, who did not endorse TBI for several years before symptom onset (41, 42). There is an albeit weak, but increased, relative risk of developing epilepsy in veterans of the recent wars in Iraq and Afghanistan who suffered TBI (43). Indeed, previous DWI studies have similarly found changes in white matter tracts in PTE patients, and tractography is increasingly showing use in identifying causes of epilepsy in otherwise non-lesional traditional imaging studies (24, 26, 44). Despite this most likely interpretation, we recognize the finding of disrupted white matter tracts in this study cannot establish cause and effect in PTE.

## Concluding Remarks

This is the first study to uniquely diagnose and further localize SHE through correlation of changes using a combination study modalities. In particular, we localize a likely acquired seizure nidus using white matter tractography in a case where traditional epilepsy imaging techniques could not identify a lesion source. Our findings are consistent with previously described presumptions as to lesion localization in SHE. However, this study is limited as a case report and by the relatively narrow literature on acquired SHE, particularly in regards to prevalence and pathophysiology. The patient reported his TBI history to the best of his ability; however, precise details are limited due to a lack of posteriori knowledge of reporting these events to a medical provider. Due to this limited history, we recognize the etiology of the development of this white matter nidus could theoretically predate injuries. Nevertheless, we demonstrate the use of white matter tractography in the diagnosis of a focal epilepsy. Further studies of patients with acquired SHE, and SHE in the general population, are necessary to describe the pathogenesis, and aid in creation of sound diagnostic criteria that may aide providers in early identification. The use of white matter tractography MRI can also be systematically studied to aid in the diagnosis of non-lesional epilepsy disorders, to better understand pathophysiology of the disease.

## Ethics Statement

This study was carried out in accordance with the recommendations of the Walter Reed National Military Medical Center’s (WRNMMC) Institutional Review Board (IRB) with written informed consent from all subjects. All subjects gave written informed consent in accordance with the Declaration of Helsinki. The protocol was approved by the WRNMMC IRB.

## Author Contributions

All authors contributed to the conception or design or the acquisition, analysis, or interpretation of data for the work, drafting or revising it critically for important intellectual content, and final approval of the version to be published, and agreed to be accountable for all aspects of the work.

## Conflict of Interest Statement

The authors declare that the research was conducted in the absence of any commercial or financial relationships that could be construed as a potential conflict of interest.

## Supplementary Material

Video S1 Correlating video of a single stereotyped sleep-related hypermotor epiplepsy episode recorded during subject’s sleep study.
