# Supplementary figures and images for: Acquired Sleep-Related Hypermotor Epilepsy with Disrupted White Matter Tracts Assessed by Multishell Diffusion Magnetic Resonance Imaging
Source: Front Neurol. 2018 Jan 22;9:6. doi: 10.3389/fneur.2018.00006 (PMC5786569; doi:10.3389/fneur.2018.00006)

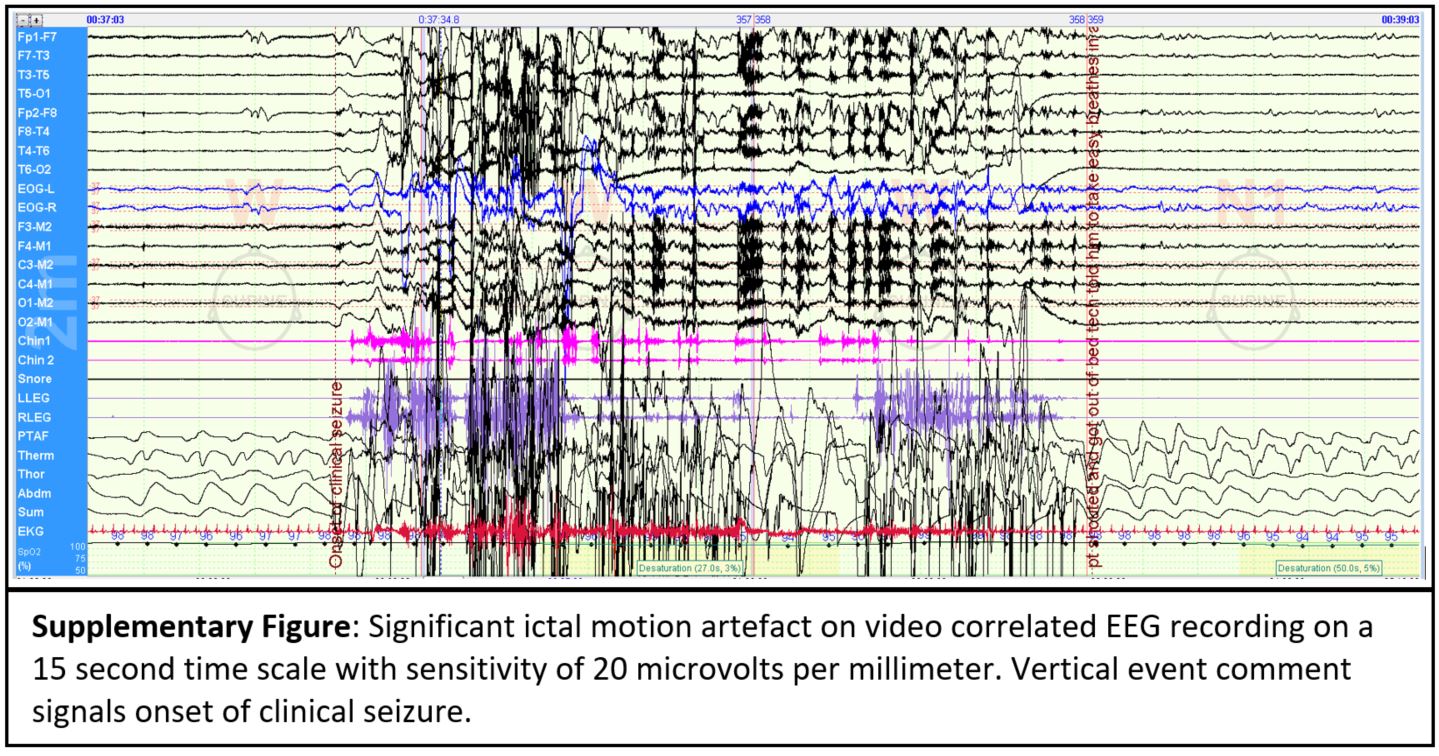

Supplement: Supplementary file 1 [file Image_1.JPEG]
